# Role of miRNA-671-5p in Mediating Wnt/β-Catenin-Triggered Podocyte Injury

**DOI:** 10.3389/fphar.2021.784489

**Published:** 2022-01-17

**Authors:** Chunhong Wang, Jiafeng Liu, Xiaoyao Zhang, Qiyan Chen, Xiaoyan Bai, Xue Hong, Lili Zhou, Youhua Liu

**Affiliations:** ^1^ Division of Nephrology, National Clinical Research Center of Kidney Disease, State Key Laboratory of Organ Failure Research, Nanfang Hospital, Southern Medical University, Guangzhou, China; ^2^ Department of Pathology, University of Pittsburgh School of Medicine, Pittsburgh, PA, United States

**Keywords:** podocyte injury, Wnt, β-catenin, miRNA-671-5p, WT1, proteinuria

## Abstract

Podocyte injury and proteinuria are the most common features of glomerular disease, which is the leading cause of end-stage renal failure. Hyperactivated Wnt/β-catenin signaling is closely associated with podocyte injury, but the underlying mechanisms are incompletely understood. Here we show that miRNA-671-5p (miR-671-5p) plays a crucial role in mediating β-catenin-triggered podocyte injury by targeting Wilms tumor 1 (WT1). Microarray-based expression profiling revealed that miR-671-5p was the most upregulated miRNA in podocytes after β-catenin activation. MiR-671-5p was colocalized with β-catenin in the glomeruli of proteinuric CKD *in vivo*. Bioinformatics analyses and luciferase reporter assays confirmed that miR-671-5p targeted WT1 mRNA. Overexpression of miR-671-5p mimics inhibited WT1 and impaired podocyte integrity, whereas miR-671-5p antagomir preserved the expression of WT1 and other podocyte-specific proteins under basal conditions or after β-catenin activation. In mouse remnant kidney model, overexpression of miR-671-5p aggravated podocyte injury, worsened kidney dysfunction and exacerbated renal fibrosis after 5/6 nephrectomy. In contrast, miR-671-5p antagomir alleviated podocyte injury and attenuated proteinuria and renal fibrotic lesions after glomerular injury *in vivo*. These studies underscore a pivotal role of miR-671-5p in mediating WT1 depletion and podocyte injury induced by β-catenin. Targeting miR-671-5p may serve as a new approach to prevent podocyte injury and proteinuria in proteinuric CKD.

## Introduction

Podocyte injury is a major pathological feature of many glomerular diseases such as focal segmental glomerulosclerosis (FSGS), IgA nephropathy (IgAN) and diabetic kidney disease (DKD) (Reiser and Sever, 2013; Fogo, 2015; Assady et al., 2017). As an integral component of the glomerular filtration barrier, podocytes and their foot processes play an essential role in preventing against proteinuria (Pavenstadt et al., 2003; Greka and Mundel, 2012). Increasing evidence demonstrates that podocyte injury not only leads to an impaired glomerular filtration and development of proteinuria, but also is instrumental in causing glomerular sclerosis in proteinuric chronic kidney disease (CKD) (Patrakka and Tryggvason, 2009; Mathieson, 2011). As podocytes are highly specialized, terminally differentiated cells, it is very challenging to restore and repopulate them once they are lost or dysfunctional (Brinkkoetter et al., 2013; Grahammer et al., 2013; Perico et al., 2016; Djudjaj and Boor, 2019). Therefore, it is of great importance to identify the extracellular culprits and delineate the molecular mechanism underlying podocyte damage.

Wnt/β-catenin is an evolutionarily conserved signaling that plays an imperative role in regulating embryonic development, injury repair and organ fibrosis (Angers and Moon, 2009; MacDonald et al., 2009; Clevers and Nusse, 2012). In many proteinuric CKD such as FSGS, DKD and IgAN, dysregulated activation of β-catenin is evident in the glomerular podocytes (Angers and Moon, 2009; MacDonald et al., 2009; Clevers and Nusse, 2012), suggesting its potential involvement in podocyte injury. Several studies have revealed that activation of β-catenin in podocytes down-regulates Wilms’ tumor 1 protein (WT1) and induces the expression of β-catenin downstream target genes such as Snail1, matrix metalloproteinase-7 (MMP-7) and components of the renin-angiotensin system (RAS) (Zhou et al., 2015a; Zhou and Liu, 2015). This leads to podocyte dedifferentiation and mesenchymal transition, which impairs podocyte integrity and disrupts glomerular filtration barrier and causes proteinuria (Matsui et al., 2007; Li et al., 2008; Dai et al., 2009; Heikkila et al., 2010; Kato et al., 2011; Garcia de Herreros and Baulida, 2012; Tan et al., 2019).

As a master transcription factor, WT1 plays a fundamental role in establishing podocyte phenotype and integrity by controlling the expression of a variety of podocyte-specific genes. Our earlier studies have shown that WT1 and β-catenin antagonize each other and competitively bind to the common transcriptional coactivator, the cyclic AMP response element binding protein (CREB) binding protein (CBP) (Zhou et al., 2015b). Activation of β-catenin has no effect on the expression of WT1 mRNA but reduces its protein expression (Zhou et al., 2015b), suggesting that β-catenin inhibition of WT1 occurs at the post-transcriptional level. We further show that β-catenin can induce the ubiquitin-mediated degradation of WT1, but this only partially accounts for the decline of WT1 protein upon β-catenin activation (Zhou et al., 2015b). These findings insinuate that some other unidentified mechanisms may be involved in mediating the loss of WT1 by β-catenin.

MicroRNAs (miRNAs) are endogenous, small single-stranded non-coding RNAs with approximately 22 nucleotides. MiRNA can bind to the 3′-untranslated region (3′-UTR) of its target mRNA and lead to the inhibition of its translation process or directly lead to the degradation of mRNA, thereby inhibiting the expression of the target genes at the protein level (Trionfini and Benigni, 2017; Ishii et al., 2020). MiRNAs play important roles in various biological processes such as organogenesis, cell proliferation and apoptosis, and the pathogenesis of human diseases (Standart, 2007; Mitchell et al., 2008; Bartel, 2009; Inui et al., 2010; Tijsen et al., 2010; Wang et al., 2010). Along this line, we hypothesized that β-catenin may down-regulate WT1 protein by regulating miRNAs in glomerular podocytes.

In this study, we conducted a microarray assay to profile miRNA expression in mouse podocytes after β-catenin activation. We found that miR-671-5p was the top hit among the most differentially expressed miRNAs in β-catenin-overexpressed podocytes. We show that miR-671-5p specifically targets WT1 and inhibits its expression, thereby impairing podocyte integrity. Therefore, targeting miR-671-5p may be a novel strategy in the treatment of proteinuric CKD.

## Methods

### Cell Culture and Treatment

Human embryonic kidney 293T cells were obtained from the American Type Culture Collection (ATCC) (Manassas, VA) and cultured in DMEM medium supplemented with 10% fetal bovine serum (FBS) at 37°C with 5% CO_2_. The conditionally immortalized mouse podocyte cell line (MPC5) was provided by Peter Mundel (Massachusetts General Hospital, Boston, MA) and maintained as described previously (Dai et al., 2009; Wang et al., 2011). To propagate podocytes, cells were cultured at 33°C with 5% CO_2_ in RPMI1640 medium supplemented with 10% FBS and 10 units/ml mouse recombinant IFN-γ (R&D Systems, Minneapolis, MN) to enhance the expression of a thermosensitive T antigen. To induce differentiation, podocytes were grown under nonpermissive conditions at 37°C with 5% CO_2_ in the absence of IFN-γ. For some studies, 293T cells or podocytes were transiently transfected with miR-671-5p mimics, inhibitor, and their respective controls (Genepharma, Shanghai, China) and/or N-terminally truncated β-catenin vector (pDel-β-cat) or psiCHECK-2-wide type/mut-WT1 by using Lipofectamine 2000 reagent (Invitrogen, Carlsbad, CA). Whole-cell lysates were prepared and subjected to real-time PCR and Western blot analyses. Cells were also subjected to immunofluorescence staining and microRNA microarray analysis.

### MicroRNA Microarray and Bioinformatics Analysis

MPC5 cells were transiently transfected with expression vector encoding constitutively activated β-catenin (pDel-β-cat) or empty vector (pcDNA3) for 24 h (*n* = 3) and then total RNA was extracted by using TRIzol (Invitrogen, Carlsbad, CA). MiRCURY LNA microRNA chips (v. 8.0, Exiqon, Vedbaek, Denmark) were used to profile the differences for miRNA expression between two groups. The candidate target genes of miR-671-5p were predicted using TargetScan program (http://www.targe tscan.org).

### Luciferase Reporter Assays

The 3′-UTR of WT1 was obtained from mouse genomic DNA by PCR and cloned into the psiCHECK-2 vector (Promega, Madison, WI) and then verified by sequencing. To test the binding specificity, the sequences in the mouse WT1 3′-UTR interacting with the miR-671-5p seed sequence were mutated from GCTTCCA to ATGGTTC. For the luciferase reporter assay, the reporter constructs were co-transfected with miR-671-5p mimic or negative control (NC) into 293T cells using lipofectamine 2000. At 24 h after transfection, luciferase activity was measured using a Dual-Luciferase report assay system (Promega), according to the manufacturer’s instructions.

### Real-Time Quantitative RT-PCR

Total RNA was isolated from cultured cells or whole kidney lysates using a TRIzol-based RNA isolation protocol (Invitrogen). For miRNA detection, RNA was reverse transcribed using the TaqMan microRNA Reverse Transcription Kit (Applied Biosystems, Foster City, CA), and then TaqMan microRNA Assay for mmu-miR-671-5p/U6 was used for PCR according to the manufacturer’s instructions. For mRNA, first-strand cDNA synthesis was carried out using 2 μg of RNA in 20 μl of reaction buffer by using a Reverse Transcription System Kit (Promega). Real-time quantitative RT-PCR (qRT-PCR) was performed using a SYBR Select Master Mix (ABI) on an ABI PRISM 7000 Sequence Detection System (Applied Biosystems) as described previously (Zhou et al., 2015b). The mRNA levels of various genes were calculated after normalizing with β-actin. The sequences of the primer pairs in qRT-PCR were as follows: mouse WT1, 5′-CAT​CCA​GGC​AGG​AAA​GTG​T-3′ and 5′-TGC​AGT​CAA​TCA​GGT​GTG​CT-3′; mouse CTGF, 5′-CAA​AGC​AGC​TGC​AAA​TAC​CA-3′ and 5′-GGCCAA ATGTGTCTTCCAGT-3′; mouse TGF-β1, 5′-GCA​ACA​TGT​GGA​ACT​CTA​CCA​GAA-3′ and 5′-GAC​GTC​AAA​AGA​CAG​CCA​CTC​A-3′; mouse β-actin, 5′-CAG​CTG​AGA​GGG​AAA​TCG​TG-3′ and 5′-CGT​TGC​CAA​TAG​TGA​TGA​CC-3′.

### Animal Models

All animals were obtained from the Southern Medical University Animal Center (Guangzhou, China) and housed in a standard environment with a regular light/dark cycle and free access to water and chow. Animal studies were approved by the Animal Ethics Committee at the Southern Medical University. For the 5/6 nephrectomy (5/6NX) model, two thirds of the left kidney of the male CD-1 mice (8 weeks) were removed through surgical resection of the upper and lower poles (week -1). One week later (week 0), the entire right kidney was removed via a right back incision. Sham-operated mice had their poles of left kidney (week -1) and right renal artery (week 0) identified, manipulated but not resected (Leelahavanichkul et al., 2010; Yang et al., 2010). Mice were randomly divided into three groups (*n* = 5 in each group): 1) sham control; 2) 5/6NX mice injected with pcDNA3 vector; and 3) 5/6NX mice injected with pCMV-pri-miR-671-5p plasmid. Plasmids were administered via hydrodynamics-based tail vein injection with a dosage of 1 mg/kg at week 2, 3, 4 and 5, respectively. At week 6, all mice were euthanized, and urine, blood and kidney tissue collected for various analyses.

For assessing the therapeutic effect of antimiR-671-5p, we utilized Adriamycin (ADR) nephropathy model, which developed robust glomerular injury, proteinuria and renal fibrotic lesions in BALB/c mice (He et al., 2011). Briefly, male BALB/c mice (6 weeks) were administered by a single intravenous injection of ADR (doxorubicin hydrochloride; Sigma-Aldrich, St. Louis, MO) at 11.5 mg/kg body weight. Oligonucleotides targeting miR-671-5p (miR-671-5p antagomir) or control antagomir were purchased from Genepharma (Shanghai, China) and injected into mice via the tail vein at 50 μg per mouse per day for 7 days. At week 2, all mice were euthanized, and urine, blood and kidney tissue collected for various analyses.

### Urinary Albumin and Creatinine Assay

Serum creatinine levels were measured by an automatic chemistry analyzer (AU480; Beckman Coulter, Pasadena, CA). Urinary albumin was measured by using a mouse Albumin ELISA Quantitation kit, according to the manufacturer’s protocol (Bethyl Laboratories, Inc., Montgomery, TX). Urinary creatinine was determined by a routine procedure as described previously (Zhou et al., 2013). Urinary albumin was standardized to creatinine and expressed as mg/mg urinary creatinine.

### *In Situ* Hybridization

Paraffin sections (2 μm) were used to assess miR-671-5p expression in the kidneys of the patients with proteinuric CKD or 5/6NX mice. *In situ* hybridization (ISH) for miR-671-5p transcripts was performed using Enhanced Sensitive ISH Detection kit Ⅱ (AP) (Boster) and digoxigenin-labeled LNA-miR-671-5p probes (Exiqon, Vedbaek, Denmark), according to the manufacturer’s protocol. Human biopsy sections were obtained from diagnostic renal biopsies performed at Nanfang Hospital. All studies involving human kidney sections were approved by the Institutional Ethics Committee at the Nanfang Hospital.

### Histology Assessment

Mouse kidney tissues were embedded in paraffin and then routinely proceeded. Quantitation was carried out on the sections stained with Periodic acid-Schiff (PAS) reagents as follows (Raij et al., 1984): grade 0, no mesangial expansion and glomerular hypertrophy; grade 1, 2, 3 and 4, mesangial expansion and glomerular hypertrophy up to 25%, 25–50%, 50–75% and 75–100%, respectively. The glomeruli in each stained section (at least 20 glomeruli) were evaluated under ×40 magnification and results averaged for each kidney. The sclerosis index for each mouse was calculated as follows: (N1 × 1 + N2 × 2 + N3 × 3 + N4 × 4)/n, where N1, N2, N3, and N4 represent the number of glomeruli graded as 1, 2, 3, and 4, respectively, and n represents the number of glomeruli assessed.

The assessment of kidney interstitial fibrosis was performed on the sections stained with Masson’s trichrome staining (MTS) under an OLYMPUS BX43 microscope equipped with a digital camera. About 10 nonoverlapping images under high-powered (400×) fields per section were randomly captured, positive areas of MTS staining were quantified by a computer-aided pointing counting technique as described previously (Mo et al., 2017). MTS-positive area (percentage of whole kidney area except tubular lumens) was assessed for collagen deposition in the kidney. Similar methods were used to quantify the fibronectin expression in the kidney sections.

### Immunohistochemical Staining

Immunohistochemical staining was performed using the established protocol (He et al., 2012). Antibodies used were as follows: rabbit anti-fibronectin (F3648; Sigma), mouse anti-α-SMA (ab7817; Abcam), mouse anti-β-catenin (610154, BD Biosciences) and rabbit anti-β-catenin (ab15180, Abcam). After incubation with the primary antibodies at 4°C overnight, slides were then stained with Biotin-SP-conjugated secondary antibody (Jackson ImmunoResearch Laboratories). Images were captured by using OLYMPUS BX43 microscope equipped with a digital camera.

### Immunofluorescence Staining and Confocal Microscopy

Kidney cryosections (3 μm thickness) were fixed with 4% paraformaldehyde for 15 min at room temperature. MPC5 cultured on coverslips were fixed with cold methanol: acetone (1:1) for 10 min at room temperature. After blocking with 10% donkey serum for 1 h, slides were immunostained with the following antibodies: ZO-1 (402200; Invitrogen), nephrin (20R-NP002; Fitzgerald Industries International), vimentin (D21H3; Cell Signaling Technology, Danvers, MA) and podocalyxin (AF1556; R&D Systems). The slides were then stained with Cy3-or Cy2-conjugated secondary antibody (Jackson ImmunoResearch Laboratories), and nuclei were stained with DAPI (4′,6-diamidino-2-phenylindole) (Sigma-Aldrich). Slides were viewed under a fluorescence microscope (Leica DMi8, Wetzlar, Germany) equipped with a digital camera.

### Western Blot Analysis

Protein expression was analyzed by Western blot analysis of whole kidney lysates or whole cell lystaes as described previously (Zhou et al., 2014). The primary antibodies used were as follows: anti-podocalyxin (AF1556; R&D Systems), anti-ZO-1 (402200; Invitrogen), anti-nephrin (ab58968; Abcam), anti-WT1 (sc-393498; Santa Cruz Biotechnology), anti-fibronectin (F3648; Sigma), anti-α-SMA (ab5694; Abcam), anti-collogen Ⅰ (BA0325, Boster Biotechnology), anti-α-tubulin (RM2007; Ray Antibody Biotech) and anti-GAPDH (RM2002; Ray Antibody Biotech). Relative protein levels of Western blots were quantified with densitometries, analyzed by ImageJ software and reported after normalizing to the loading controls. Relative protein levels over the control group (setting as 1.0) were reported.

### Statistical Analyses

All data examined are expressed as mean ± SEM. Statistical analyses were performed using SPSS 19.0 (SPSS Inc., Chicago, IL). Comparison between groups was made using *t* test or one-way ANOVA followed by Student-Newman-Kuels test or Dunnett’s T3. *p* < 0.05 was considered significant.

## Results

### MiR-671-5p Is Induced in Podocytes After β-Catenin Activation and Specifically Targets WT1

**FIGURE 1:**
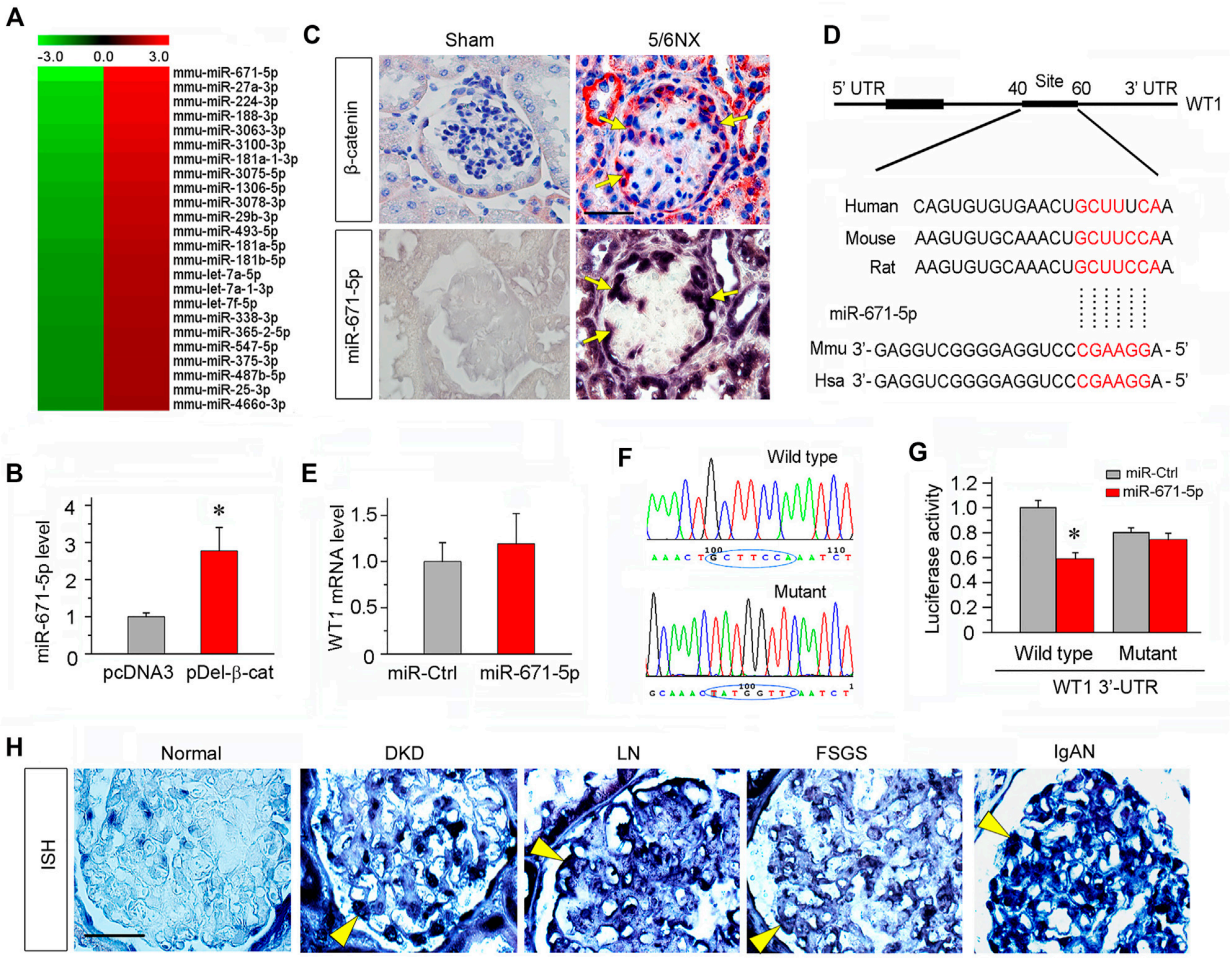
miR-671-5p is induced by β-catenin in podocytes and targets WT1. **(A)** Microarray chip analysis of miRNAs expression in different groups (left: pcDNA3 control group; right: pDel-β-cat group). The red and green colors indicated high or low expression, respectively. **(B)** Mouse podocytes (MPC5) were transfected with empty vector (pcDNA3) or β-catenin expression plasmid (pDel-β-cat) for 24 h. qRT-PCR analysis showed the expression of miR-671-5p in different groups. *p < 0.05 (*n* = 3). **(C)** Co-localization of β-catenin and miR-671-5p in glomerular podocytes of diseased kidney. Kidney serial sections (3 μm) of 5/6NX mice were subjected to immunostaining for β-catenin and *in situ* hybridization for miR-671-5p. Representative micrographs from sham group are also shown. Arrows indicate positive staining in podocytes. Scale bar, 20 µm. **(D)** Bioinformatics analysis shows the predicted binding sites of miR-671-5p in the WT1 3′-untranslatd region (UTR) using the TargetScan software. **(E)** qRT-PCR analysis shows that overexpression of miR-671-5p did not affect WT1 mRNA level in mouse podocytes. MPC5 cells were transfected with miRNA negative control (miR-Ctrl) or miR-671-5p mimics (miR-671-5p) for 24 h. **(F)** Sequence validation of the wild type or mutant WT1 3′-UTR for the luciferase reporter construction. The wild-type miR-671-5p binding site in WT1 3′-UTR **(upper)** and the mutated one **(bottom)** in the region corresponding to the miR-671-5p seed sequence are shown. **(G)** Luciferase reporter assay show that miR-671-5p mimics decreased the luciferase activity in 293T cells co-transfected with wild-type WT1 3′ UTR, but not with mutant WT1 3′ UTR. *p < 0.05. **(H)** Representative micrographs show miR-671-5p expression in glomerular podocytes of human kidney biopsies from the patients with various CKDs by *in situ* hybridization. Arrowheads indicate the positive staining for miR-671-5p in glomerular podocytes. Kidney tissues adjacent to renal cell carcinoma from patients who underwent carcinoma resection were used as normal control. Scale bar, 20 µm.

To investigate the potential role of miRNAs in mediating Wnt/β-catenin-induced podocyte injury, we used an unbiased approach to profile miRNA expression in podocytes after β-catenin activation. To this end, mouse podocytes were transfected with constitutively activated β-catenin expression vector (pDel-β-cat) or empty vector (pcDNA3) and then subjected to microRNA array analysis. We found substantial changes in the miRNA expression after β-catenin activation, with 119 miRNA up-regulated and 91 down-regulated. The relative expression level of these miRNAs is presented as a heatmap (Figure 1A). The miR-671-5p was identified as the most differentially expressed miRNA induced by β-catenin in podocytes. This finding was further confirmed *in vitro* by quantitative, real-time PCR (qPCR). As shown in Figure 1B, consistent with the results of miRNA microarray, miR-671-5p was upregulated in podocytes after transient transfection of pDel-β-cat plasmid. *In situ* hybridization (ISH) revealed that miR-671-5p specifically upregulated in the glomerular podocytes of diseased kidney induced by 5/6NX, as compared with sham controls. Co-staining for β-catenin and miR-671-5p on serial sections demonstrated that β-catenin colocalized with miR-671-5p (Figure 1C), suggesting a role for β-catenin in upregulating miR-671-5p expression *in vivo* after podocyte injury.

Bioinformatics analyses using miRNA target prediction tools such as TargetScan revealed that miR-671-5p could target WT1, as the 3′-untranslatd region (UTR) of WT1 harbored the conserved site complementary to the seed sequence of miR-671-5p (Figure 1D). We found that transfection of mouse podocytes (MPC5) with miR-671-5p mimic did not affect WT1 mRNA expression (Figure 1E), suggesting that miR-671-5p may regulate WT1 expression at the post-transcriptional level.

To determine whether WT1 is a direct target of miR-671-5p, the luciferase reporter plasmids containing WT1 3′-UTR (wild type) or mutant sequence corresponding to the miR-671-5p seed sequence were constructed (Figure 1F), and transfected into 293T cells, in combination with miR-671-5p mimic or control miRNA (miR-Ctrl). As shown in Figure 1G, transfection with miR-671-5p mimic inhibited the luciferase activity of the reporter containing wild-type WT1 3′-UTR but not the mutant WT1 3′-UTR, suggesting that WT1 is a direct target of miR-671-5p.

### MiR-671-5p Is Up-Regulated in Glomerular Podocytes in Human CKD

To investigate the clinical relevance of miR-671-5p to the pathogenesis of human proteinuric CKD, we performed ISH for detecting miR-671-5p with a digoxigenin-labeled LNA probes in human kidney biopsies from the patients with various proteinuric CKDs. Kidney biopsies from diabetic kidney disease (DKD), lupus nephritis (LN), focal segmental glomerulosclerosis (FSGS) and IgA nephropathy (IgAN) were subjected to ISH for miR-671-5p, whereas non-tumor kidney tissue sections from renal cell carcinoma patients who underwent cancer resection were used as normal control. As shown in Figure 1H, miR-671-5p was barely detectable in normal kidney, but markedly induced in the glomerular podocytes in different proteinuric CKDs. These results indicate a close association between miR-671-5p and the pathogenesis of podocyte injury in human CKD.

### MiR-671-5p Targets WT1 and Impairs Podocyte Integrity *In Vitro*

**FIGURE 2:**
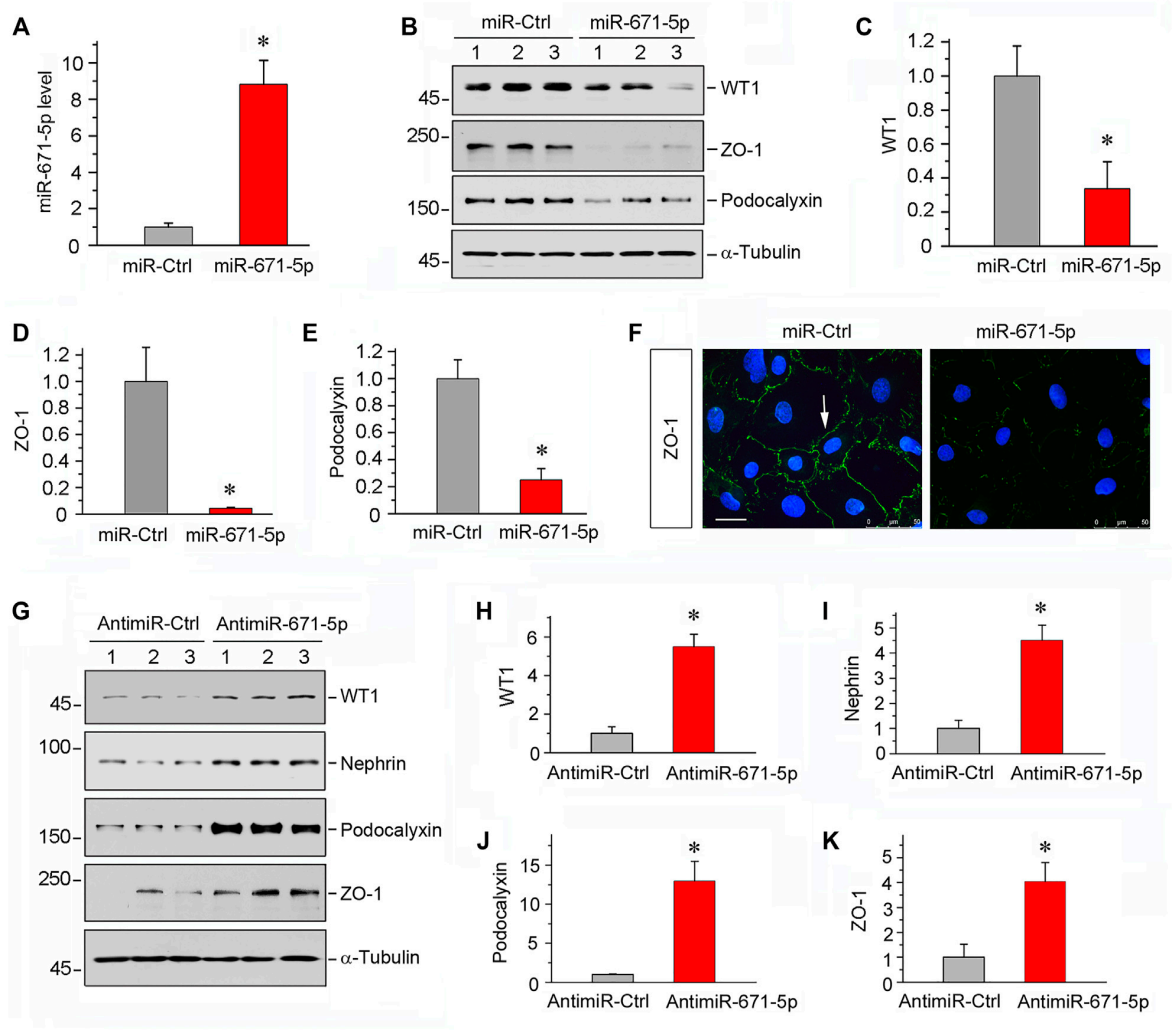
Overexpression of miR-671-5p impairs but knockdown of miR-671-5p protects podocyte integrity *in vitro*. Mouse podocytes (MPC5) were transfected with miR-671-5p mimics (miR-671-5p) or negative control (miR-Ctrl) for 24 h. **(A)** qRT-PCR analysis shows the relative levels of miR-671-5p after transfection. *p < 0.05 (*n* = 3). **(B–E)** Representative Western blot **(B)** and graphic presentations of WT1 **(C)**, ZO-1 **(D)** and podocalyxin **(E)** were presented. *p < 0.05 (*n* = 3). **(F)** Representative micrographs show the expression and distribution of ZO-1 in podocytes after miR-671-5p overexpression. Scale bar, 50 µm. **(G–K)** Inhibition of miR-671-5p protects podocyte integrity. MPC5 cells were transfected with miR-671-5p inhibitor (AntimiR-671-5p) or control (AntimiR-Ctrl) or for 24 h. Representative Western blot **(G)** and graphic presentations of WT1 **(H)**, nephrin **(I)**, podocalyxin **(J)** and ZO-1 **(K)** were presented. *p < 0.05 (*n* = 3).

To study the potential role of miR-671-5p in podocyte biology, we maneuvered miR-671-5p expression in mouse podocytes (MPC5) by transfecting either miR-671-5p mimic or inhibitor. As shown in Figure 2A, transfection with miR-671-5p mimic markedly increased miR-671-5p level, as assessed by qPCR analysis. We found that overexpression of miR-671-5p substantially inhibited WT1 protein expression in MPC5 cells (Figures 2B,C), suggesting that miR-671-5p can inhibit its target gene as expected. Interestingly, inhibition of WT1 by miR671-5p down-regulated podocalyxin and ZO-1 expression (Figures 2B,D,E). Similar results were obtained by immunostaining for ZO-1 protein (Figure 2F). In contrast, inhibition of miR671-5p by anti-miRNA oligonucleotides (antimir-671-5p) in MPC5 cells up-regulated WT1, nephrin, podocalyxin and ZO-1 (Figures 2G–K). These findings suggest that miR-671-5p specifically targets WT1 and impairs podocyte integrity.

### MiR-671-5p Aggravates β-Catenin-Induced Podocyte Injury *In Vitro*

**FIGURE 3:**
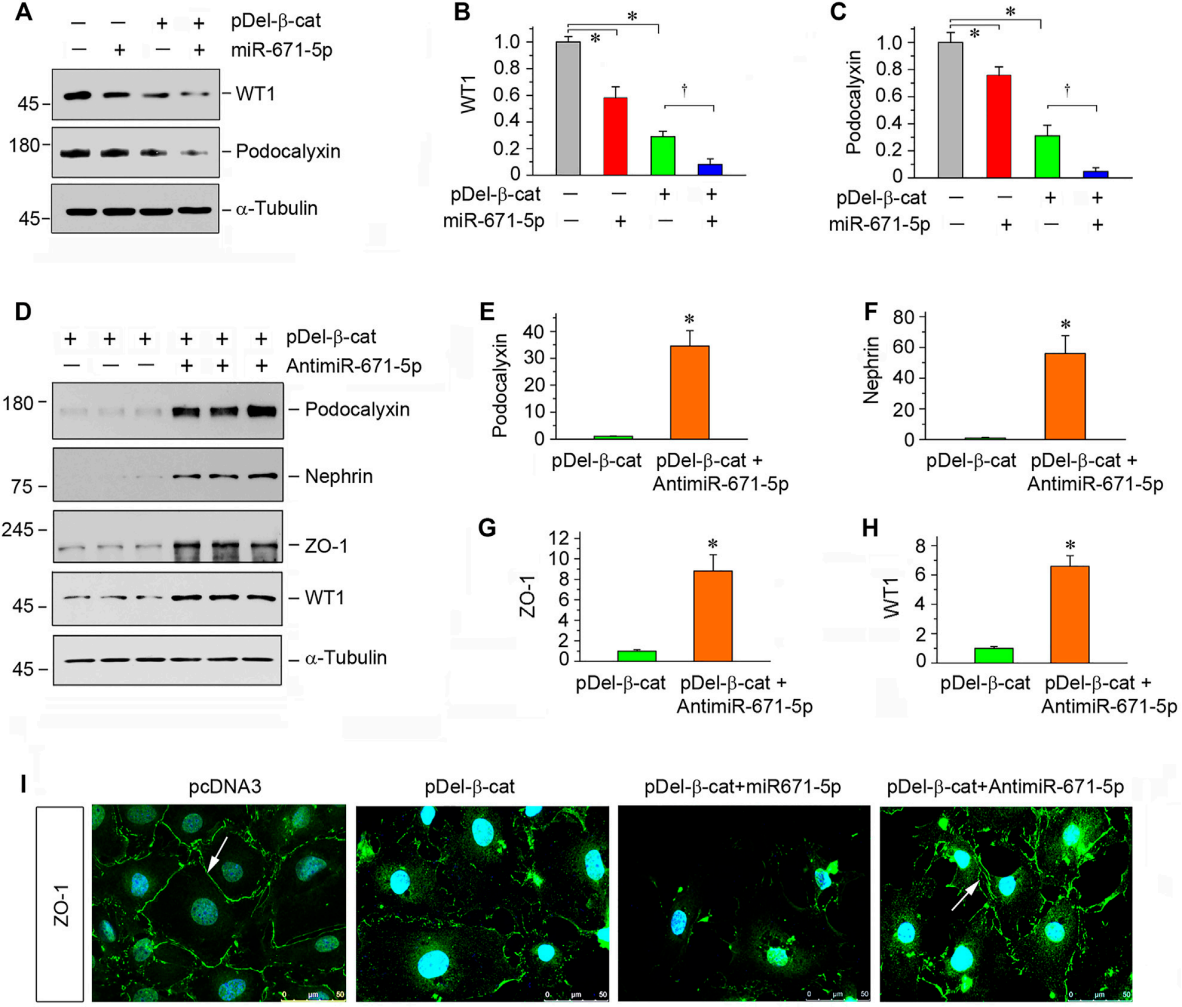
miR-671-5p aggravates β-catenin-induced podocyte injury while miR-671-5p inhibitor ameliorates it *in vitro*. **(A–C)** Representative Western blot **(A)** and graphic presentations of WT1 **(B)** and podocalyxin **(C)** were presented. MPC5 cells were transfected with miR-671-5p mimics (miR-671-5p) or/and β-catenin expression plasmid (pDel-β-cat) for 24 h *p < 0.05 versus pcDNA3 controls; †*p* < 0.05 versus pDel-β-cat (*n* = 3). **(D–H)** Representative Western blot **(D)** and graphic presentations of podocalyxin **(E)**, nephrin **(F)**, ZO-1 **(G)** and WT1 **(H)** were presented. MPC5 cells were transfected with β-catenin expression plasmid (pDel-β-cat) or/and miR-671-5p inhibitor (AntimiR-671-5p) for 24 h *p < 0.05 (*n* = 3). **(I)** Representative micrographs show the expression of ZO-1 in different groups as indicated. MPC5 cells were transfected with β-catenin expression plasmid (pDel-β-cat) and miR-671-5p mimics (miR-671-5p)/miR-671-5p inhibitor (AntimiR-671-5p) for 24 h, respectively. Scale bar, 50 µm.

To validate the role of miR-671-5p in mediating β-catenin-induced podocyte injury, we transfected β-catenin expression plasmid (pDel-β-cat), along with miR-671-5p mimic or miR-671-5p inhibitor, into mouse podocytes. Earlier studies show that transfection with pDel-β-cat plasmid induces Snail1 and plasminogen activator inhibitor 1 (PAI-1) expression in podocytes (Zhou et al., 2015b), confirming its ability to stimulate β-catenin downstream genes. As illustrated in Figures 3A–C, overexpression of either β-catenin or miR-671-5p inhibited WT1 and podocalyxin. Moreover, combination of β-catenin and miR-671-5p led to further suppression of WT1 and podocalyxin (Figures 3A–C), suggesting that miR-671-5p aggravates β-catenin-induced podocyte injury. However, transfection with miR-671-5p inhibitor upregulated podocalyxin, nephrin, ZO-1 and WT1 expression, even in the presence of β-catenin activation (Figures 3D–H). Immunofluorescence staining for ZO-1 gave rise to similar results (Figure 3I).

### MiR-671-5p Accelerates 5/6NX-Induced Podocyte Injury and Glomerulosclerosis *In Vivo*

**FIGURE 4:**
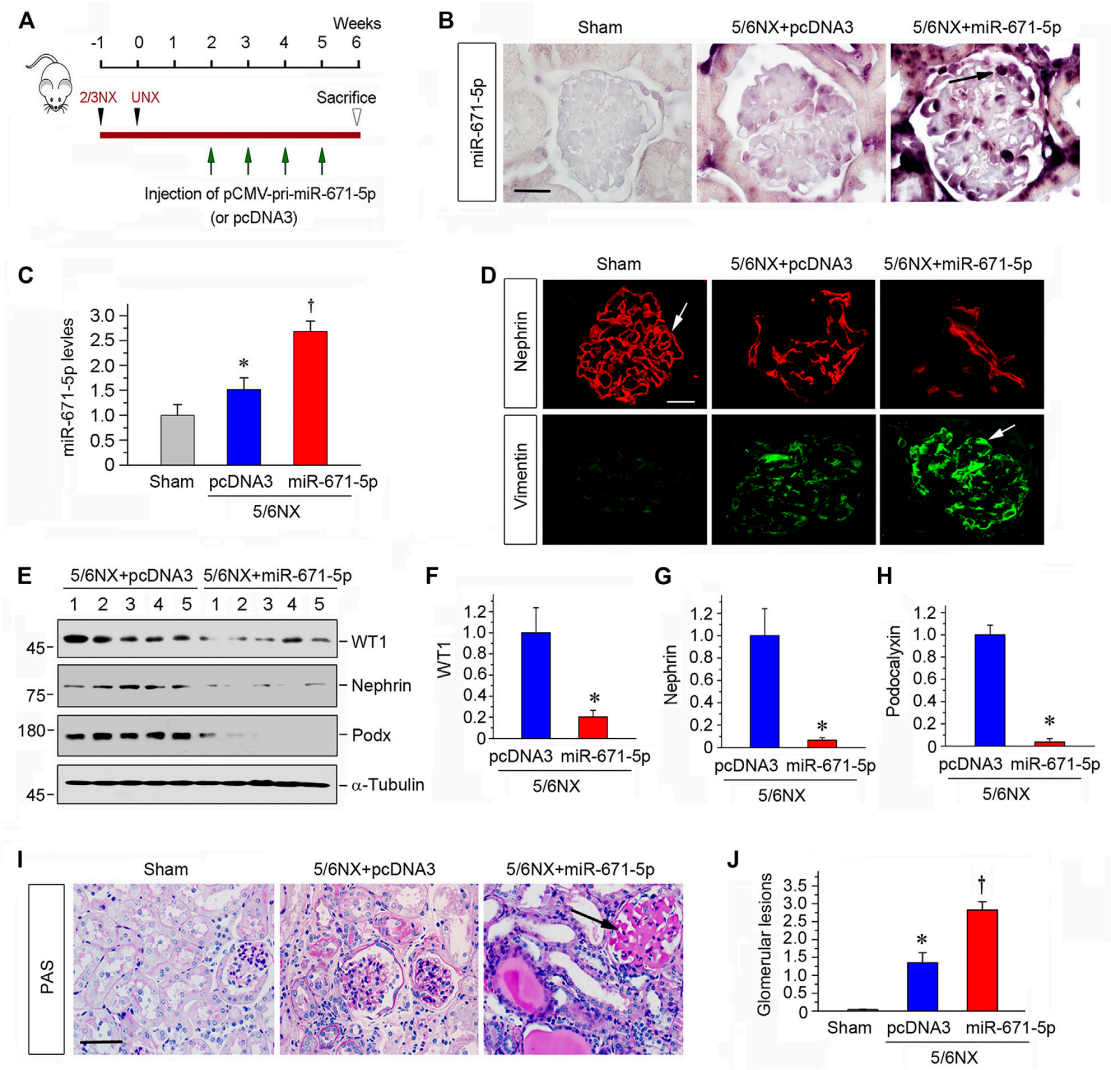
Ectopic expression of miR-671-5p accelerates podocyte injury and glomerulosclerosis in 5/6NX model. **(A)** Experimental design. Black Arrowheads indicate the time of kidney resection. Green arrows indicate the injections of pCMV-pri-miR-671-5p or pcDNA3 plasmid. **(B)** Representative micrographs show miR-671-5p expression in glomerular podocytes in 5/6NX model by *in situ* hybridization. Scale bar, 20 µm. **(C)** qRT-PCR analysis of miR-671-5p levels in different groups as indicated. *p < 0.05 versus sham controls; †*p* < 0.05 versus 5/6NX alone (*n* = 5–6). **(D)** Immunofluorescence staining show renal expression of nephrin and vimentin in different groups as indicated. Frozen kidney sections were stained for nephrin and vimentin. Scale bar, 20 µm. **(E–H)** Representative Western blot **(E)** and graphic presentations of WT1 **(F)**, nephrin **(G)** and podocalyxin **(H)** were presented. *p < 0.05 (*n* = 5–6). **(I)** Representative micrographs show periodic acid-Schiff (PAS) staining of the kidneys in different groups. Scale bar, 50 µm. **(J)** Quantitative determination of glomerular lesions (based on PAS staining) in different groups. *p < 0.05 versus sham controls, †*p* < 0.05 versus 5/6NX (*n* = 5–6).

The finding on the effect of miR-671-5p on podocyte injury *in vitro* prompted us to investigate its potential effect on proteinuric kidney disease *in vivo*. To this end, we used a mouse model of CKD induced by 5/6NX, characterized by progressive podocyte injury, glomerulosclerosis and loss of renal function (Xiao and Liu, 2013). As presented in Figure 4A, pCMV-pri-miR-671-5p plasmid or pcDNA3 plasmid were administered via tail vein for 4 times, starting from 2 weeks after 5/6NX surgery (Figure 4A). As shown in Figures 4B,C, miR-671-5p level was increased in 5/6NX group compared with sham controls, and injections of pCMV-pri-miR-671-5p plasmid further increased miR-671-5p level. ISH revealed that miR-671-5p was mainly expressed in glomerular podocytes (Figure 4B).

We next assessed podocyte injury by examining the expression of nephrin and vimentin. As shown in Figure 4D, immunofluorescence staining exhibited that overexpression of miR-671-5p accelerated the loss of nephrin and further increased glomerular vimentin expression in 5/6NX mice. Western blot analysis also showed that overexpression of miR-671-5p suppressed the expression of WT1, nephrin and podocalyxin in this model (Figures 4E–H). PAS staining showed that 5/6 NX caused mild glomerular hypertrophy and matrix deposition, capillary collapse and tubular dilation with expanded lumen, and overexpression of miR-671-5p markedly worsened these pathological lesions (Figure 4I). The extent of glomerular lesions was assessed by semi-quantitative analysis and presented in Figure 4J. These data illustrate that miR-671-5p aggravates podocyte injury and glomerular sclerotic lesions.

### MiR-671-5p Aggravates Kidney Dysfunction and Exacerbates Renal Fibrosis *In Vivo*

**FIGURE 5:**
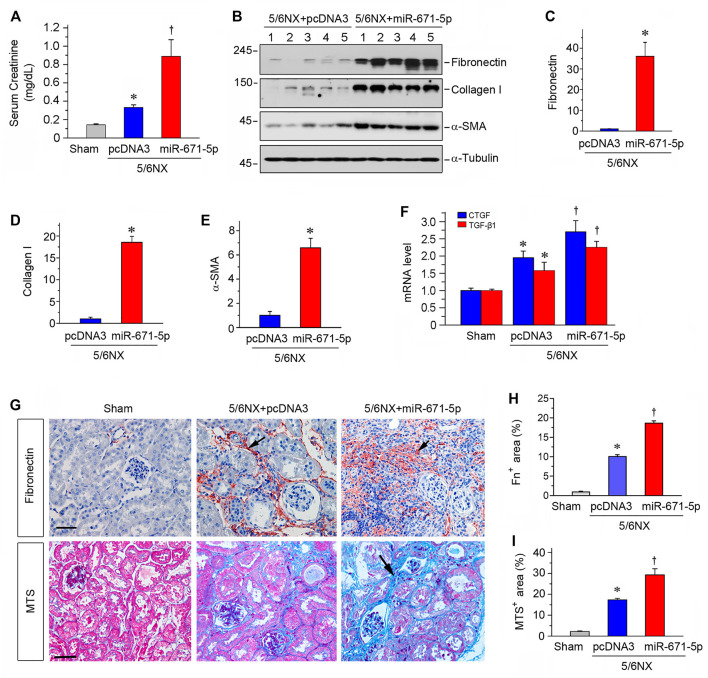
Expression of miR-671-5p *in vivo* aggravates kidney dysfunction and fibrosis in 5/6NX model. **(A)** Expression of miR-671-5p *in vivo* aggravates kidney dysfunction in 5/6NX mice. Serum creatinine was assessed in different groups as indicated. *p < 0.05 versus sham controls, †*p* < 0.05 versus 5/6NX (*n* = 5–6). **(B–E)** Representative Western blots **(B)** and graphic presentations of fibronectin **(C)**, collagen I **(D)** and α-SMA **(E)** were presented. *p < 0.05 (*n* = 5–6). **(F)** qRT-PCR analysis shows CTGF and TGF-β1 mRNA levels in different groups. *p < 0.05 versus sham controls, †*p* < 0.05 versus 5/6NX (*n* = 5–6). **(G)** Representative micrographs show that overexpression of miR-671-5p aggravated fibronectin deposition and fibrotic lesions in the 5/6NX kidneys. Paraffin kidney sections were stained for fibronectin (upper panel) and subjected to Masson’s trichrome staining (MTS) (bottom panel). Scale bar, 50 µm. **(H,I)** Quantitative determination of renal fibronectin expression **(H)** and renal collagen deposition (based on MTS) **(I)** in different groups. *p < 0.05 versus sham controls, †*p* < 0.05 versus 5/6NX (*n* = 4–6).

We further assessed kidney function by measuring serum creatinine level. As shown in Figure 5A, serum creatinine level was elevated after 5/6NX, and expression of miR-671-5p further increased serum creatinine in this model. We found that overexpression of miR-671-5p also increased the expression of numerous fibrosis-related proteins such as fibronectin, collagen I and α-smooth muscle actin (α-SMA), as demonstrated by Western blot analyses of whole kidney lysates (Figures 5B–E). Consistently, qPCR showed that miR-671-5p induced the mRNA expression of TGF-β1 and connective tissue growth factor (CTGF) in 5/6NX mice (Figure 5F).

We further assessed the fibrotic lesions in the 5/6NX kidneys after overexpression of miR-671-5p. As shown in Figures 5G,H, 5/6NX induced the deposition of fibronectin in kidneys, and overexpression of miR-671-5p aggravated the deposition. Masson’s trichrome staining (MTS) also revealed significant collagens deposition in the kidneys after 5/6NX, and overexpression of miR-671-5p further increased their deposition (Figures 5G,I). Taken together, these results indicate that miR-671-5p aggravates renal fibrotic lesions in 5/6NX mice *in vivo*.

### Inhibition of MiR-671-5p Ameliorates Podocyte Injury and Renal Fibrosis in ADR Nephropathy

**FIGURE 6:**
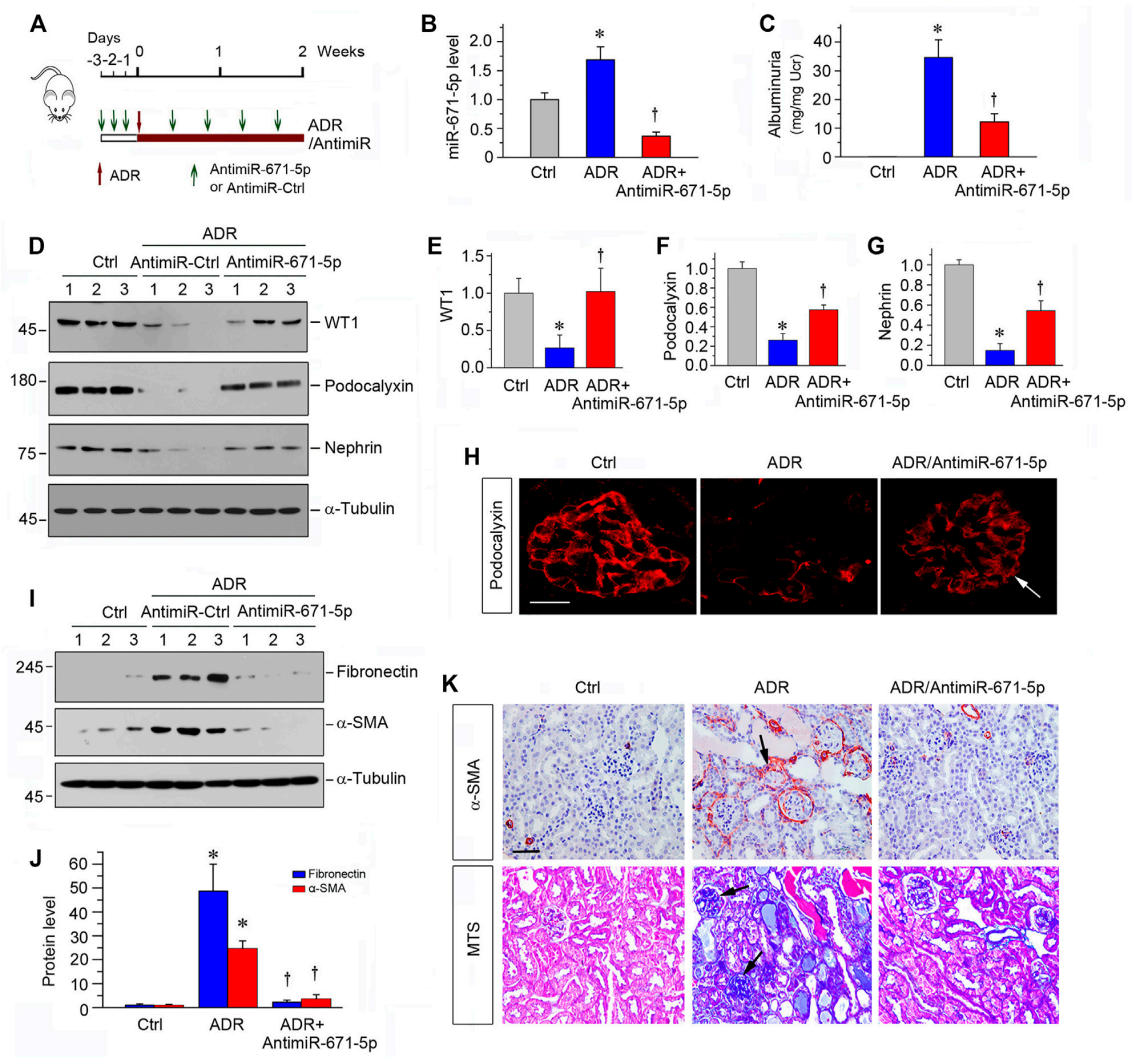
Inhibition of miR-671-5p reduces proteinuria and renal fibrotic lesions in ADR nephropathy. **(A)** Experimental design. Red Arrows indicate the time of ADR injection. Green arrows indicate the different time points of antagomir injections. **(B)** qRT-PCR analysis shows that miR-671-5p level was increased in ADR group compared with control, and injections of antimiR-671-5p decreased miR-671-5p level. *p < 0.05 versus normal controls; †*p* < 0.05 versus ADR (*n* = 5–6). **(C)** Inhibition of miR-671-5p reduces proteinuria in ADR nephropathy. Urinary albumin levels were assessed in mice at 2 weeks after ADR injection and expressed as mg/mg creatinine. *p < 0.05 versus normal controls; †*p* < 0.05 versus ADR (*n* = 5–6). **(D–G)** Representative Western blots **(D)** and graphic presentations of WT1 **(E)**, podocalyxin **(F)** and nephrin **(G)** were presented. *p < 0.05 versus normal controls, †*p* < 0.05 versus ADR alone (*n* = 5–6). **(H)** Immunofluorescence staining shows that antimiR-671-5p preserved renal podocalyxin expression in ADR nephropathy. Arrow indicate positive staining. Scale bar, 20 µm. **(I,J)** Representative Western blots **(I)** and graphic presentations of fibronectin and α-SMA **(J)** were presented. *p < 0.05 versus normal controls, †*p* < 0.05 versus ADR alone (*n* = 5–6). **(K)** Representative micrographs show that antimiR-671-5p inhibited α-SMA expression (upper panel) and renal fibrotic lesions (bottom panel) in different groups as indicated. Scale bar, 50 µm.

To further confirm the role of miR-671-5p in proteinuric CKD, we used another mouse model of podocyte injury and proteinuria induced by ADR, a model of human FSGS (Pippin et al., 2009). As shown in Figure 6A, ADR was administered at day 0, and miR-671-5p antagomir was injected intravenously at different time points as indicated. The experiments were terminated at 2 weeks after ADR injection. As illustrated in Figure 6B, renal miR-671-5p level was reduced by miR-671-5p antagomir in this model. Urinary albumin levels were elevated at 2 weeks after ADR injection, and antimiR-671-5p largely abolished albuminuria in this model (Figure 6C). We then assessed the level of WT1 protein, the target of miR-671-5p. As shown in Figures 6D,E, ADR reduced WT1 expression, whereas antimiR-671-5p largely restored its level. Furthermore, antimiR-671-5p restored the expression of podocalyxin and nephrin, which were down-regulated by ADR (Figures 6D,F,G). Immunofluorescence staining also showed that antimiR-671-5p restored podocalyxin level and distribution (Figure 6H). Taken together, it appears that miR-671-5p plays a role in podocyte injury by targeting WT1, and antimiR-671-5p restores WT1, thereby preserving podocyte integrity.

We also assessed the renal fibrotic lesions in this ADR nephrology model. As shown in Figures 6I,J, renal fibronectin and α-SMA were markedly induced after ADR, and antimiR-671-5p abolished the induction of these proteins (Figures 6I,J). Similarly, immunostaining for α-SMA and Masson’s trichrome staining demonstrated that antimiR-671-5p ameliorated myofibroblast activation and mitigated renal fibrotic lesions in ADR nephropathy (Figure 6K).

## Discussion

In this study, using an unbiased microarray expression profiling approach, we have identified miR-671-5p as a key downstream effector of Wnt/β-catenin signaling, which mediates podocyte injury by targeted inhibition of WT1. This conclusion is supported by several lines of evidence. First, miR-671-5p is induced in podocytes after β-catenin activation *in vitro* and colocalizes with β-catenin in glomerular podocytes *in vivo*, and it is specifically upregulated in glomerular podocytes of human kidney biopsies from patients with proteinuric CKD. Second, miR-671-5p targets the 3′-UTR of WT1 and inhibits its expression at the post-transcriptional level. Third, overexpression of miR-671-5p mimic impairs podocyte phenotype and integrity, whereas miR-671-5p antagomir preserves podocyte integrity under basal conditions or after β-catenin activation. Finally, overexpression of miR-671-5p *in vivo* aggravates podocyte injury, glomerulosclerosis and renal fibrotic lesions in 5/6NX mice, while miR-671-5p antagomir ameliorates podocytopathy and renal fibrotic lesions after glomerular injury. These studies underscore a pivotal role of miR-671-5p in mediating podocyte injury and glomerular lesions in proteinuric CKD. Our findings also uncover the intimate interplay among β-catenin, miR-671-5p and WT1, and provide novel insights into the mechanism how Wnt/β-catenin activation triggers podocyte dysfunction, proteinuria and glomerulosclerotic lesions.

Podocytes are highly specialized and terminally differentiated cells, with unique and sophisticated 3-dimensional (3D) structure characterized by foot processes and slit diaphragm (Greka and Mundel, 2012). Such a fine 3D structure of podocytes is largely controlled by WT1, a master transcription factor exclusively expressed in glomerular podocytes in the adult kidney. WT1 controls the expression of a host of podocyte-specific proteins such as nephrin and podocalyxin (Palmer et al., 2001; Guo et al., 2004; Wagner et al., 2004; Lowik et al., 2009; Rachel E.; Wang et al., 2011; Dong et al., 2015). Extensive studies have demonstrated that loss of WT1 is a common feature of podocytopathy that occurs in virtually all forms of proteinuric CKD in animal models and humans (Jian-Kan Guo et al., 2002; Niaudet and Gubler, 2006; Zhou et al., 2015b). Our earlier studies have shown that WT1 and β-catenin functionally antagonize each other in podocytes. Under normal physiological conditions, WT1 is highly expressed and β-catenin is minimal and inactivated (Zhou et al., 2015b). As such, WT1 is dominant over β-catenin in normal glomeruli, which keeps podocyte healthy and fully differentiated. However, in the pathological state, β-catenin is activated whereas WT1 is lost, thereby making β-catenin a predominant regulator in controlling gene transcription (Zhou et al., 2015b). The mutual antagonism between β-catenin and WT1 appears to be mediated through diverse mechanisms. We previously show that β-catenin and WT1 competitively bind to the common transcriptional coactivator CBP in a mutually exclusive manner and thus antagonize each other functionally (Zhou et al., 2015b). In addition, activation of β-catenin also induces ubiquitin-mediated degradation of WT1 protein, providing another means for β-catenin to negatively control WT1 protein in podocytes (Zhou et al., 2015b). Here our present study indicates that by inducing miR-671-5p, β-catenin constrains WT1 protein by the miRNA-mediated inhibition at the post-transcriptional level. Collectively, it is conceivable that β-catenin restrains WT1 activities by three distinctive mechanisms, including competitive binding to CBP, ubiquitin-mediated protein degradation and miRNA-based inhibition.

The findings in the present study could have significant clinical implications, as miR-671-5p is also induced in glomerular podocytes of human kidney biopsies from patients with various CKDs such as FSGS, IgAN, DKD and lupus nephritis (LN) (Figure 1). Because these CKDs share several common pathological features characterized by podocyte lesions and proteinuria, it is plausible to speculate that induction of miR-671-5p could represent a convergent response of podocytes, which results in WT1 depletion and podocyte injury. *In situ* hybridization reveals that induction of miR-671-5p in podocytes is common among different proteinuric CKDs, indicating a close association between miR-671-5p and the pathogenesis of human glomerular lesions. This observation is of significance, as miRNAs are not always evolutionally conserved across different species and even conserved miRNAs do not necessarily display the same expression levels or patterns at different stages within a species (Ha et al., 2008; Chen et al., 2020). The comparable induction pattern of miR-671-5p suggests an evolutionally conserved response of glomerular podocytes after injury in mice and humans.

The present study demonstrates that controlling miR-671-5p expression by different maneuvers may be an effective approach to preserve WT1 protein, thereby ameliorating proteinuric CKD. Inhibition of miR-671-5p via antagomir not only preserves WT1 and podocyte integrity under basal conditions, but also ameliorates β-catenin-induced podocyte lesions by restoring WT1 expression *in vitro* (Figures 2, 3). In contrast, overexpression of miR-671-5p decrease WT1 and podocyte-specific proteins such as nephrin and podocalyxin. Furthermore, miR-671-5p appears to work in concert with β-catenin to further aggravate podocyte lesions. The function of miR-671-5p is further confirmed in mouse models of proteinuric CKD induced by 5/6NX and ADR, which are characterized by podocyte injury, proteinuria and glomerulosclerosis. Consistently, overexpression of miR-671-5p accelerates 5/6NX-induced podocyte injury and renal insufficiency, whereas miR-671-5p antagomir restores WT1 and prevents the progression of ADR nephropathy. Notably, inhibition of miR-671-5p also mitigates kidney interstitial fibrosis. This is probably a consequence secondary to the alleviation of podocyte injury and proteinuria. However, we cannot exclude the possibility that inhibition of miR-671-5p may have direct beneficial effects on tubular epithelial cells, as its expression is also induced in injured tubules (Figure 1). Regardless of the mechanisms, the results from the present study provide the proof-of-principle that inhibition of miR-671-5p could be a promising approach for developing therapeutics to treat proteinuric CKDs.

It should be pointed out that, apart from miR-671-5p, there are other miRNAs that may also participate in repressing WT1 protein and cause proteinuria. For example, miR-193a has been reported to target WT1 for inhibition, thereby leading to the pathogenesis of FSGS (Gebeshuber et al., 2013). A recent study has shown that miR-466o-3p also plays a role in mediating Wnt/β-catenin-triggered WT1 repression in podocytes in mice, but not in humans. As such, the clinical relevance of miR-466o-3p to human CKDs remains to be determined. The fact that multiple miRNAs are involved in WT1 regulation in podocytes is not surprising, as many miRNAs targeting the same mRNA is a common feature of miRNA action. On the other hand, bioinformatics analyses show that at least 152 genes are potentially controlled directly by miR-671-5p. Functional and pathway enrichment analyses reveal that miR-671-5p may play an important role in podocyte injury by other mechanisms beyond WT1 inhibition. Therefore, the relative contributions of each miRNA to WT1 suppression in podocytes *in vivo*, as well as the larger landscape of signaling around miR-671-5p remains elusive and deserves further investigation.

In summary, we show herein that miR-671-5p is upregulated in podocytes after β-catenin activation and colocalizes with β-catenin in podocytes of diseased kidneys. We show that miR-671-5p specifically targets the 3′-UTR of WT1 mRNA and inhibits WT1 expression. Overexpression of miR-671-5p reduces WT1 protein and impairs podocyte phenotype and integrity, whereas miR-671-5p antagomir maintains podocyte integrity after β-catenin activation both *in vitro* and *in vivo*. Although more studies are needed, our findings suggest that targeting miR-671-5p may serve as a new approach to prevent against podocyte injury and proteinuric CKDs.

## Data Availability

The original contributions presented in the study are included in the article/Supplementary Materials, further inquiries can be directed to the corresponding authors.
